# Differences in Achilles tendon stiffness in people with gout: a pilot study

**DOI:** 10.1186/s12891-020-03598-3

**Published:** 2020-10-07

**Authors:** Simon Otter, Catherine Payne, Anna-Marie Jones, Nick Webborn, Peter Watt

**Affiliations:** 1 School of Health Sciences, University of Brighton, 49 Darley Rd, Eastbourne, BN20 7UR UK; 2 Centre for Regenerative Medicine and Devices, University of Brighton, Lewes Road, Brighton, BN2 4AT UK; 3 School of Sport and Service Management, University of Brighton, Hillbrow, Denton Road, Eastbourne, BN20 7SR UK; 4 Research and Development, Sussex Partnership NHS Foundation Trust, Swandean, Arundel Road, Worthing, BN13 3EP UK

**Keywords:** Gout, Achilles tendon, Shear wave elastography

## Abstract

**Background:**

Gout has been associated with weaker foot/leg muscles and altered gait patterns. There is also evidence of on-going foot pain and an increased risk of tendinopathy, with the Achilles and patella tendons most frequently affected in gout. Additionally, the inflammation associated with gout may change tissue elasticity. Ultrasound imaging utilising shear wave elastography (SWE) offers a non-invasive method of quantifying changes in tendon stiffness. SWE findings have not previously been reported in individuals with gout. We sought to determine differences in Achilles tendon stiffness in people with gout compared to controls (non-gout).

**Methods:**

A cross sectional study comparing 24 people with gout and 26 age/sex-matched controls. Clinical and demographic data were collated, and US imaging used to determine tendon thickness, presence of gouty tophi and/or aggregates and levels of angiogenesis. Ten shear wave elastography (SWE) measures were taken along the centre of a longitudinal section of the mid-portion of each Achilles tendon. Prior to data collection, intra-observer error was good (>0.69). Data were summarised using descriptive statistics and a repeated measures ANCOVA was used to compare SWE measures between the two groups for the left and right foot separately after accounting for Body Mass Index (BMI).

**Results:**

A small proportion of those with gout presented with intra-tendon aggregates and/or intra-tendon tophi in one or both tendons. There was no statistically significant difference in tendon thickness between groups. Neo-vascularity was present in a third of gout participants. SWE findings demonstrated significantly reduced tendon stiffness in those with gout compared to controls: right Achilles mdiff =1.04 m/s (95% CI (0.38 to 1.7) *p* = 0.003 and left Achilles mdiff = 0.7 m/s (95% CI 0.09 to 1.32) *p* = 0.025. No relationship between the presence of tophi and SWE values were detected.

**Conclusion:**

Subjects with chronic gout show significantly reduced Achilles tendon stiffness compared to non-gout controls. From a clinical standpoint, our findings were similar to SWE measurements in subjects with Achilles tendinopathy and who did not have gout.

## Background

Gout occurs as a result of a metabolic inefficiency to fully metabolise purines and is considered to be the most common inflammatory arthritis [1, 2]. The resultant production of highly insoluble monosodium urate crystals located in joints and tendons causes a range of symptoms [1, 2]. Gout has an increasing prevalence both in the UK and worldwide [1–4]. While estimates vary, up to 6.8% of some populations are affected [3]. Gout is associated with acute attacks of severe joint pain and swelling, particularly affecting the foot [4]. Pain and impairment often do not entirely normalise after a gout flare-up has subsided [5], leading to general and foot-specific pain and disability, reduced ankle joint angular velocity and resulting in altered walking patterns [6–8]. Taken together, pain, time off work, limitations in ones’ ability to move or undertake exercise and subsequent loss of participation in social activities all reduce quality of life for those with gout [6, 9, 10].

The Achilles tendon (AT) is a common location for the deposit of monosodium urate crystals, forming subcutaneous palpable bodies known as tophi [11]. These structures are located within the tendon and will affect the structural arrangement of the tendon fibres impacting on the mechanical properties and function of the AT [12]. In gout, there are also measurable signs of inflammation in the AT (e.g. intra tendinous power Doppler signals) [13]. Inflammatory markers are generally considered to be a hallmark of rheumatic disorders such as gout. Inflammatory processes alter the tissue composition, increasing water content, and can result in changes to tissue structure and tissue stiffness (or elasticity). While the AT is the strongest tendon in the body, it is frequently injured, and the pathology of those injuries is heterogeneous [14, 15]. The mechanical properties of tendons are key to the transmission of muscle tension to the skeleton, elastic energy storage and to limiting load stress on muscles [16]. Consequently, measurement of the structural properties of the tendon is an important part of understanding the changes that may occur and affect the background pathogenesis [17]. Clinicians commonly use ultrasound imaging (US) to visualise tendon structure in AT disorders, since the technique is minimally invasive, quick and often easily available for use [18]. Shear wave elastography (SWE) is a non-invasive and dynamic method of quantifying changes in tendon elasticity, involving application of a focused ultrasound pulse to the tissue being imaged [19–21]. SWE is a type of US elastography that uses shear waves generated by a remote radiation force of focused ultrasonic beams to assess tissue elasticity. It measures the velocity of shear waves (in metres per second) moving through tissue, and displays these in a quantitative manner via a real-time elastogram, in which elasticity is portrayed as a colour-coded representation over a B-mode image. In SWE images stiffer tissues are red, while softer tissues are blue [22–24]. Importantly in ultrasonography, previous work indicates SWE is more independent of user skill than other modes of US imaging [25]. A systematic review of SWE as a diagnostic tool indicated good correlation with conventional ultrasound results, MRI and clinical examination [26]. More recently, SWE per se has received attention with regard to conditions involving tendons. Compared to other methods commonly used, it has been shown to be a simple way to measure tendon stiffness and identify pathology with good precision and increased diagnostic accuracy [27]. A review of imaging modalities in gout has highlighted the value of ultrasound for monitoring monosodium urate deposition and damage associated with inflammation, but SWE was not reported upon [28]. We aimed to utilise SWE to identify differences in Achilles tendon stiffness in people with gout compared to controls (non-gout). We hypothesised that stiffness of the AT would be reduced by the inflammatory processes and the consequences of monosodium urate crystal deposits affecting the structure and function of the AT.

## Methods

### Participants and setting

The terminology used for gout used in the paper follows the G-CAN consensus statements [29]. In this cross sectional, case-controlled study, participants with gout were recruited from an out patient podiatry clinic in East Sussex. Ethical approval was granted by the University of Brighton (17–009) and Office for Research Ethics Committees Northern Ireland (ORECNI) (232339). The inclusion criteria (Table 1) were those diagnosed with gout (aged 25–80) by their GP with no self-reported Achilles tendinosis. Most patients with gout are managed in the primary care setting and the diagnosis for gout is usually clinical with identification of monosodium urate crystals and/or serum urate measurements not generally undertaken [3]. Consequently, the clinical presentation of gout was checked according to the 2015 ACR/EULAR classification criteria [30], ensuring the clinical presentation met the clinical criteria. Non-gout participants were simultaneously recruited from the same patient population, typically with the same co-morbidities as those with gout, and met the same exclusion criteria. Inclusion/exclusion criteria for both groups are detailed in Table 1.

**Table 1:** Inclusion/Exclusion criteria

Gout participants	Non-gout (control) participants
Inclusion criteria	Inclusion criteria
Current diagnosis gout No Current or recent (last 6 months) Achilles tendonosis	No diagnosis of gout No Current or recent (last 6 months) Achilles tendonosis
Exclusion criteria	Exclusion criteria
Current acute gout flare Previous Achilles tendon rupture Self-reported current or recent (last 6 months) Achilles tendinosis Surgery to the Achilles tendon Concomitant inflammatory arthritis e.g. rheumatoid arthritis Concomitant neuromuscular disease Significant foot deformity (e.g. Charcot foot) Participants taking fluoroquinolone antibiotics Those unable to lie on an examination couch Active foot ulceration	Previous Achilles tendon rupture Self-reported current or recent (last 6 months) Achilles tendinosis Surgery to the Achilles tendon Concomitant inflammatory arthritis e.g. rheumatoid arthritis Concomitant neuromuscular disease Significant foot deformity (e.g. Charcot foot) Participants taking fluoroquinolone antibiotics Those unable to lie on an examination couch Active foot ulceration

A clinician identified potential participants over an 18-month period (July 2018–December 2019) during their normal podiatry appointment, based on presenting complaint and/or medical history. If agreeable, potential participants were provided with an information pack and contacted after 48 h by the chief investigator (SO) to answer questions and arrange an appointment if they wished to opt in to the study. Data were collected during a single session outside of participants’ normal appointment time. Informed, written consent was obtained prior to data collection. We sought to recruit 25 people in each group. As SWE has not been carried out in those with gout previously, a formal power calculation was not possible. However, research focusing on ultrasound imaging of the AT alone in those with gout used a similar number of participants and found significant differences between populations [13].

### Data collection

Baseline demographic details were captured together with comorbidities and key potential confounding factors including BMI and self-reported levels of exercise using the validated relative physical activity question [31].

### Ultrasound scanning techniques

All measurements were taken with a Siemens ACUSON S2000™ Helix Evolution Ultrasound system (Siemens Medical Solutions, Malvern, PA, USA) ultrasound imaging system, utilising a pre-programmed AT setting. The AT is a favoured site for assessment as it meets the parameters required for SWE, while avoiding common variables that lead to limitations with US imaging [32]. The AT was selected as it offers:

- Close proximity target tissue (AT) to the transducer (within 3 cm)
- Largely homogeneous tissue
- No structures present to dampen shear stress measurement (e.g. large vessels)
- Distance between tissue boundaries
- Ease of identifying anatomical location

Previously compression elastography ultrasound measurements have been limited by poor reproducibility [33]. Therefore a standardised protocol for SWE ultrasound assessment was adopted, based on validated studies where Inter-observer reliability varied between κ0.87–1 [13] and ICC between operators was 0.80 [34].

#### Conventional ultrasound technique

All US images were captured by an experienced operator (SO) with 7 years ultrasound imaging experience. A 40 mm 14 L5 linear probe was used to visualise the AT length from the insertion of the AT over the calcaneus to the lowest fibres of the gastrocnemius. Scanning for pathological features of gout were undertaken in transverse and longitudinal axis. All SWE measures were taken in a longitudinal axis between 40 mm and 80 mm proximal to the insertion of the AT, capturing the mid-portion of the tendon where tendon pathology is most frequently encountered [35, 36]. For SWE measures a 40 mm 9LT linear probe was utilised. To maintain standardisation, the following uniform US settings to optimise imaging were used:

- Greyscale US settings 15 MHz probe, 15 dB, DR60
- Elastography US settings 9Mhz probe, 6 dB, DR65

(where: MHz = probe frequency, dB = decibels, DR = dynamic range (gain))

Throughout, participants were invited to lie prone with their feet relaxed freely overhanging an examination couch in line with previous validated protocols [34].

#### Intra-observer reliability US image interpretation

To assess intra-observer agreement of US characteristics, reliability was determined using (ICC (2)) with measures captured from three control subjects using 10 images on two separate occasions. The anonymous images were used to calculate reliability. Greyscale US measurements (tendon thickness) varied between 3.25–3.63 mm and SWE measures 9.27–10. Intra-observer error (ICC (2)) values were consistently between 0.69 and 0.707. Intra-observer error values of 0 to 0.2 are considered poor, 0.2 to 0.4 fair, 0.4 to 0.6 moderate, 0.6 to 0.8 good and 0.8 to 1.0 excellent [37]. We determined the mean of three measurements could used to determine tendon thickness without loss of reliability. Previously validated protocols [13] demonstrated almost perfect inter-rater reliability for those pathologies unique to gout (e.g. tophi) together with good intra-observer reliability. Therefore, subsequent US images and SWE measures were assessed by a single operator (SO) in real time.

#### Tendon structure & damage

AT thickness was measured at the thickest part of the mid-portion in a longitudinal axis. A partial tendon tear was defined as a focal discontinuity [38], whilst a complete tendon rupture was defined as a complete loss of tendon substance [39]. Tendon grading was based on a semi-quantitative system proposed by Archambault et al. [40] detailed in Table 2 and recorded bilaterally.

**Table 2:** Tendon grading definitions

Grade	Definition
1	normal-appearing tendon with homogeneous fibrillar echotexture
2	a focal fusiform swelling and/or diffuse enlarged tendon
3	a hypoechoic area within the tendon with/or without tendon enlargement

#### Features of urate deposition

Deposits of monosodium urate (MSU) or ‘tophi’; are frequently seen in gout and strongly reflect US waves. As a consequence, the presence of tophi can easily be confirmed [41]. US characteristics for tophi and aggregates were pre-determined in accordance with standardized definitions; the Outcome Measures in Rheumatology (OMERACT) group [42]. Tophus are circumscribed, inhomogeneous, hyperechoic accumulations, whereas ‘aggregates’ are heterogeneous and hyperechoic foci of uric acid deposits that maintain a high degree of reflectivity. The number of gouty tophi and/or aggregates in the AT was recorded bilaterally.

#### Features of inflammation

Tendon vascularity was assessed over the length of the tendon and defined as the presence of the power Doppler signal. A semi-quantitative scale (0–4) previously reported by Ohberg et al. [43] was used to quantify the level of neo angiogenesis present bilaterally.

#### Shear wave elastography (SWE)

**Fig. 1:**
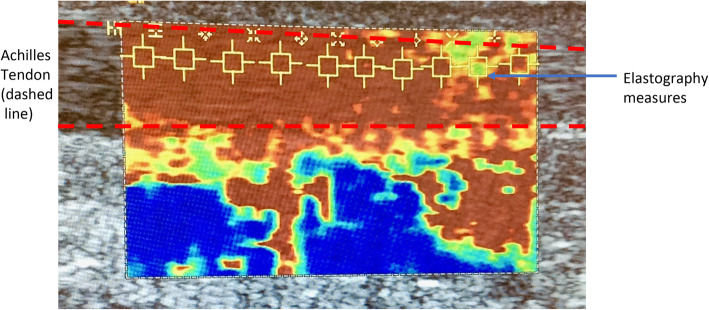
Illustration of SWE measures being undertaken

Following conventional ultrasound, the system was placed in the virtual touch IQ (VTIQ) mode. Ten real-time SWE measures between 0.5–10 m/s were taken along the centre of a longitudinal section of the mid-portion of the AT bilaterally (Fig. 1). If SWE readings > 10 m/s are present the machine reads ‘HIGH’ and the highest value of 10 m/s was recorded.

#### Data analysis

**Fig. 2:**
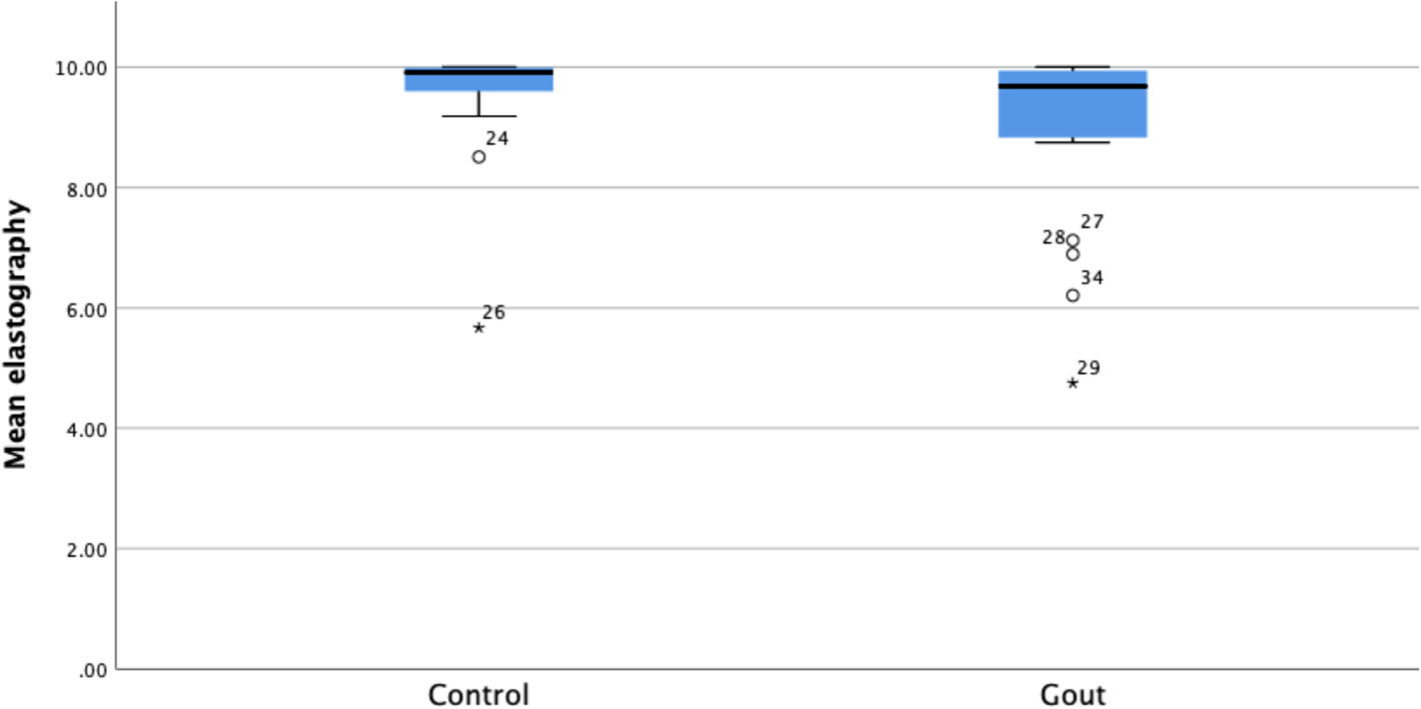
Boxplot of mean SWE values by group (gout vs non-gout)

Data were entered into SPSS and checked for accuracy, adherence to normal distribution and assumptions for inferential analysis (Fig. 2) [44]. Baseline clinical and demographic characteristics were summarised using descriptive statistics: count (n), mean, standard deviation (sd), range and percentages (%). Between-group (gout vs non-gout) comparisons were made for each characteristic, using Chi square and Student’s t-tests for categorical and continuous variables respectively. Given the within-subjects repeated-measures design used, an ANCOVA was used to compare SWE measures between the two groups for the left and right AT separately, while adjusting for covariates (Body Mass Index (BMI)). Bivariate Pearson correlation was used to determine any relationship between the number of tophi and SWE values. Statistical tests were significant for *p* < 0.05. SPSS v25 (IBM SPSS Statistics Version 25.0. Armonk, NY: IBM Corp.) used for all analyses.

## Results

In total, 40 people with gout were recruited with 24 subsequently participating (60%). Of those not taking part, 8 declined and 5 subsequently excluded, two were subsequently not contactable and one did not attend their appointment. A population of 26 age/sex matched non-gout subjects were simultaneously recruited from the same clinical population.

### Demographic and clinical data

Baseline demographic and clinical data are displayed in Table 3. Only 3 (13%) participants had experienced a gout attack within the last 3 months and 4 (17%) participants had clinically palpable gouty tophi over the foot/ankle region. Palpable subcutaneous tophi were located around the Achilles tendon for 3 subjects and on lesser digits for 1 subject. Chi square and Student’s t-tests showed that those with gout had significantly higher BMI (mdiff = 3.56 (95% CI 0.79 to 6.33; *p* = 0.013) and had a slightly higher proportion of comorbidities; but did not differ in respect of data concerning gender, ethnicity nor self-reported levels of activity.

**Table 3:** Clinical and Demographic characteristics

	Gout (*n* = 24)	Non-gout (*n* = 26)
Male/Female (n)	19/5	21/5
Mean age (SD)	70.63 (11)	72.73 (10)
Mean BMI (SD)*	30.53 (4.97)	26.98 (4.77)
Ethnicity		
Caucasian	23 (96%)	25 (96%)
Non-Caucasian	1 (4%)	1 (4%)
Mean self-reported relative physical activity (range 1–3)	1.86	1.88
Comorbidities N (%)		
Diabetes	12 (50)	11 (42)
Hypertension	18 (75)	15 (58)
Dyslipidaemia	15 (63)	13 (50)
Cardiac disease	10 (42)	6 (23)
Chronic kidney disease	8 (33)	2 (6)
Mean duration gout (SD)	9.39 (8.49) years	–
Pharmacological management gout		–
None	7 (29)	
NSAIDs	2 (8)	
Prednisolone	2 (8)	
Allopurinol	10 (42)	
Colchine	3 (13)	

### Conventional ultrasound

**Fig. 3:**
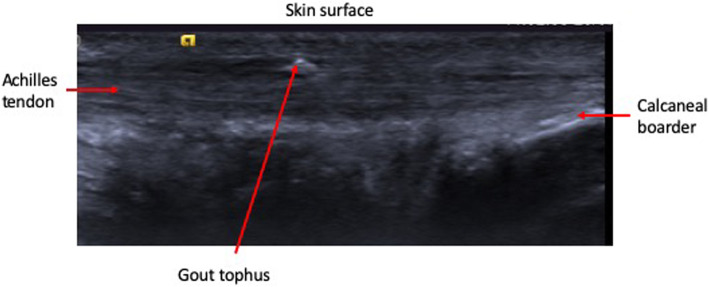
Gout tophus appearance in greyscale, conventional ultrasound

No subjects were noted to have either partial or complete tendon rupture. Table 4 highlights the main features of conventional ultrasound. There were no clinically meaningful differences in tendon grading and no significant difference in tendon thickness between groups. Small, but non-significant, differences in levels of neo-vascularity were noted between groups, with four (15%) non-gout participants exhibiting grade 1 or 2 neo-vascularity, whereas eight (33%) people with gout exhibited neo-vascularity between grades 1–3. Either gouty tophi and/or aggregates were visualised in nine (38%) participants with gout, these features being absent in the non-gout population – Fig. 3 illustrates a gout tophus on greyscale US.

**Table 4:** Ultrasound characteristics of the AT

	Gout (*n* = 24)		Non-gout (*n* = 26)	
	Right	Left	Right	Left
Tendon grade n (%)				
Grade 1	19 (80)	17 (71)	18 (50)	19 (54)
Grade 2	5 (21)	7 (30)	8 (31)	7 (27)
Grade 3	0	0	0	0
Midpoint tendon thickness mm mean (SD)	5.66 (1.51)	5.86 (1.58)	5.87 (1.46)	5.54 (0.88)
Mean number intra-tendon aggregates (range)	0.25 (0–2)	1.17 (0–7)	0	0
Mean number intra-tendinous tophi (range)	0.54 (0–5)	1.08 (0–17)	0	0
Tendon neo-vascularity n (%)				
Grade 0	19 (80)	17 (71)	23 (88)	24 (92)
Grade 1	2 (8)	4 (17)	3 (12)	1 (4)
Grade 2	2 (8)	2 (8)	0	1 (4)
Grade 3	1 (4)	1 (4)	0	0
Grade 4	0 (0)	0 (0)	0	0

### Shear wave elastography (SWE)

**Fig. 4:**
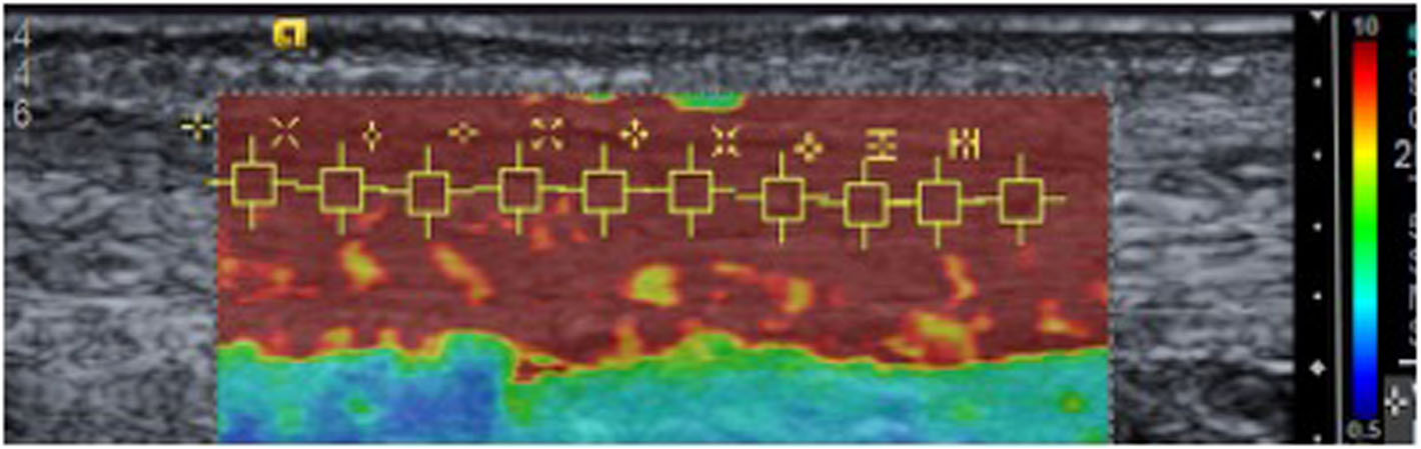
SWE scan control (non-gout) participant

**Fig. 5:**
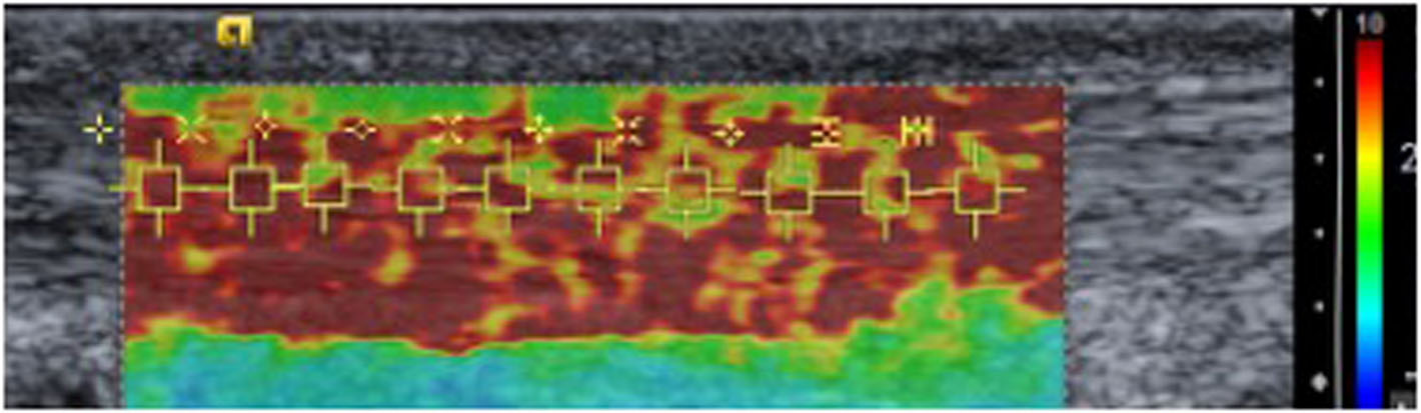
SWE scan gout participant

SWE findings shown in Table 5 demonstrated significantly lower levels of tendon stiffness in those with gout compared with the non-gout group (right AT *p* = 0.003, left AT *p* = 0.025). Figure 4 illustrates a typical SWE scan from a participant without gout, whereas Fig. 5 exemplifies the SWE pattern seen in those with gout, where the tendon is more heterogeneous throughout, with a wider range of SWE readings noted.

**Table 5:** Shear wave elastography (SWE) findings

Achilles tendon	Group	SWE readings Mean ± SD (range) m/s	Mean difference in SWE readings (95% CI)	Standard error	Differences between groups
Right	Gout (*n* = 24)	8.90 ± 1.65 (4.11–10)	1.04 (0.38–1.7)	0.33	*p* = 0.003
	Non-gout (*n* = 26)	9.76 ± 0.48 (7.31–10)			
Left	Gout (*n* = 24)	9.17 ± 1.4 (4.7–10)	0.7 (0.09–1.32)	0.31	*p* = 0.025
	Non-gout (*n* = 26)	9.66 ± 0.65 (7.05–10)			

No significant relationship between the number of tophi and SWE values were detected

## Discussion

Using shear wave elastography, we have for the first time, demonstrated that people with gout exhibit significantly lower stiffness in the AT compared with age/sex matched non-gout participants. Previously, Aubrey and colleagues [45] reported that those with mid-portion AT tendinopathy demonstrated significantly lower mean SWE velocities than did those with a non-pathological AT. The loss of Achilles tendon stiffness we identified in those with gout was often greater than that demonstrated using a similar protocol in otherwise healthy runners with known Achilles tendinopathy [34]. The mean SWE values determined by Payne et al. [34] ranged from 7.91 m/s-9.56 m/s compared with mean SWE values in our participants with gout of 4.75 m/s-10 m/s. Recent work [46] suggests Achilles tendinopathy in healthy subjects involves both thickening of the tendon and softening (as reflected in lower SWE values), however our participants with gout did not report symptoms of AT.

Tendon pathology in the lower limbs has been shown to be more frequent in those with gout than in those with either osteoarthritis or healthy controls [13, 47]. A systematic review and meta-analysis revealed that abnormal ultrasound findings (i.e. tendon thickening, increased vascularity, hypoechogenicity) in the AT were predictive of tendinopathy [48]. However in our findings from those with gout, tendon thickening did not appear to be present, nor were considerably higher intra-tendon power Doppler readings noted, possibly suggesting a different pathological model from standard tendinopathy. A previous US study [13] demonstrated a much higher prevalence of intra-tendinous tophi (73%) than in non-gout participants. MSU deposition has been reported in 15–48% of the AT using dual energy CT scanning techniques [18, 49]. Our conventional ultrasound findings were toward the upper end of these results, but not as high as Carroll et al. [13], which may reflect different population sampling strategies. Previously, Chhana et al. [12] reported that MSU crystals directly interact with tenocytes to reduce cell viability and function, which may contribute to tendon damage. We did not identify any correlation between the number of tophi and SWE measures, which may further support a different pathological model*.* None of the studies reviewed above have reported SWE measurements. However currently, the lack of agreed and validated SWE values for tendinopathy may limit its use in the formal assessment of tendon damage [50].

Previous clinical and laboratory-based studies have reported weaker foot/leg muscles in those with gout than in non-gout participants [51, 52]. This muscle weakness was also associated with greater levels of foot pain [53]. Those with gout also exhibited slower walking speeds than control participants [53]. Current international recommendations for gout management [54] highlight the importance of diet together with lifestyle changes such as increased levels of physical activity. In 2008 the UK National Institute for Health and Care Excellence (NICE) [55] produced management recommendations for osteoarthritis that focused on the lower limb and emphasized that muscle strengthening should be undertaken before increasing aerobic activity. While we did not measure muscle strength, our contention here is that those participants with considerable loss of tendon stiffness might find that some types of increased load-bearing exercise have the potential to hasten the end stages of the continuum model of tendon pathology [56].

A key strength of our work is that we recruited people from a community-based outpatient clinic, and as such our participants were broadly representative of patients with gout treated in primary care. None were experiencing symptoms of an acute gout flare, nor Achilles tendinosis, yet significant loss of tendon stiffness was demonstrated. The primary limitation of our study was that the researcher (SO) undertaking US scans and SWE readings was not blinded to participant diagnosis, which may have led to inadvertent bias. Pathologies consistent with gout when seen on ultrasound are highly characteristic, typically with excellent inter-rater reliability [13]. Consequently, it is difficult to completely blind sonographers to the underlying diagnosis, but the addition of a second sonographer may add to the reliability of SWE measures in future work. The US machine we used for SWE readings also has a limitation where a maximum SWE value of 10 m/s can be recorded, after which a value of HIGH is given. Consequently, there may be a ceiling effect to some of the measurements leading to a possible underestimation of the differences in AT stiffness. Moreover, there has been limited use of SWE to assess soft tissue stiffness in other inflammatory arthropathies, making direct comparisons difficult. For example, in Ankylosing Spondylitis significant structural impairment of the Achilles tendon was noted using sonoelastography [57]. However, rather than reporting SWE values, areas of the AT were simply colour coded into normal or pathological, making direct comparison impossible.

## Conclusions

Subjects with gout demonstrated significantly reduced Achilles tendon stiffness compared to non-gout controls. From a clinical standpoint, our SWE findings for the AT in those with gout were similar to SWE measurements in subjects with Achilles tendinopathy who did not have gout. This finding suggests the AT in people with gout may be less effective at transmitting muscle force and potentially more susceptible to further pathology and injury.

## Data Availability

The datasets generated and/or analysed during the current study are not publicly available as some identifying information is present to enable patient re-referral if required, but are available from the corresponding author on reasonable request.

## Abbreviations

AT: Achilles tendon
MSU: Monosodium urate
OMERACT: Outcome measures in rheumatology
SWE: Shear Wave Elastography
US: Ultrasongraphy
